# Core Outcomes Set for Trials in People With Coronavirus Disease 2019

**DOI:** 10.1097/CCM.0000000000004585

**Published:** 2020-08-17

**Authors:** Allison Tong, Julian H. Elliott, Luciano Cesar Azevedo, Amanda Baumgart, Andrew Bersten, Lilia Cervantes, Derek P. Chew, Yeoungjee Cho, Tess Cooper, Sally Crowe, Ivor S. Douglas, Nicole Evangelidis, Ella Flemyng, Elyssa Hannan, Peter Horby, Martin Howell, Jaehee Lee, Emma Liu, Eduardo Lorca, Deena Lynch, John C. Marshall, Andrea Matus Gonzalez, Anne McKenzie, Karine E. Manera, Charlie McLeod, Sangeeta Mehta, Mervyn Mer, Andrew Conway Morris, Saad Nseir, Pedro Povoa, Mark Reid, Yasser Sakr, Ning Shen, Alan R. Smyth, Tom Snelling, Giovanni FM Strippoli, Armando Teixeira-Pinto, Antoni Torres, Tari Turner, Andrea K. Viecelli, Steve Webb, Paula R. Williamson, Laila Woc-Colburn, Junhua Zhang, Jonathan C. Craig

**Affiliations:** 1 Sydney School of Public Health, The University of Sydney, Sydney, NSW, Australia.; 2 Centre for Kidney Research, The Children’s Hospital at Westmead, Sydney, NSW, Australia.; 3 Cochrane Australia, School of Public Health and Preventive Medicine, Monash University, Melbourne, VIC, Australia.; 4 Department of Critical Care Medicine, Hospital Sirio-Libanes, São Paulo, Brazil.; 5 College of Medicine and Public Health, Flinders University, Adelaide, Australia.; 6 Department of Medicine, Denver Health, Denver, CO.; 7 Faculty of Medicine, University of Queensland at Princess Alexandra Hospital, Brisbane, Qld, Australia.; 8 Crowe Associates Ltd, Oxon, United Kingdom.; 9 Department of Medicine, Pulmonary Sciences and Critical Care, Denver Health and University of Colorado Anschutz, School of Medicine Denver, Aurora, CO.; 10 Department of Editorial and Methods, Cochrane, London, United Kingdom.; 11 Nuffield Department of Medicine, University of Oxford, Oxford, United Kingdom.; 12 Department of Internal Medicine, School of Medicine, Kyungpook National University, Daegu, South Korea.; 13 Department of Internal Medicine, Faculty of Medicine, University of Chile, Santiago, Chile.; 14 Jonze Society, Brisbane, Qld, Australia.; 15 Department of Surgery, University of Toronto, Toronto, ON, Canada.; 16 Telethon Kids Institute, Perth, WA, Australia.; 17 Department of Infectious Diseases, Perth Children’s Hospital, Perth, WA, Australia.; 18 Department of Medicine and Interdepartmental Division of Critical Care Medicine, University of Toronto, Toronto, ON, Canada.; 19 Department of Medicine, Divisions of Critical Care and Pulmonology, Charlotte Maxeke Johannesburg Academic Hospital and Faculty of Health Sciences, University of the Witwatersrand, Johannesburg, South Africa.; 20 Department of Medicine, University of Cambridge, Cambridge, United Kingdom.; 21 Critical Care Centre, CHU Lille, and Lille University, F-59000 Lille, France.; 22 Nova Medical School, CHRC, New University of Lisbon, Polyvalent Intensive Care Unit, Sao Francisco Xavier Hospital, CHLO, Lisbon, Portugal.; 23 Center for Clinical Epidemiology and Research Unit of Clinical Epidemiology, OUH Odense University Hospital, Odense, Denmark.; 24 Department of Anesthesiology and Intensive Care, Jena University Hospital, Jena, Germany.; 25 Department of Respiratory Medicine, Peking University Third Hospital, Beijing, China.; 26 Evidence Based Child Health Group, University of Nottingham, Nottingham, United Kingdom.; 27 Department of Emergency and Organ Transplantation, University of Bari, Bari, Italy.; 28 Department of Pulmonology Hospital Clinic. University of Barcelona, CIBERES, IDIBAPS, Barcelona, Spain.; 29 Department of Biostatistics, University of Liverpool, Liverpool, United Kingdom.; 30 Section of Infectious Diseases Department of Medicine, National School of Tropical Medicine, Baylor College of Medicine, Houston, TX.; 31 Evidence-based Medicine center, Tianjin University of Traditional Chinese Medicine, Tianjin, China.

**Keywords:** clinical trial, coronavirus, critical care, infection, patients, sepsis

## Abstract

**Objectives:**

The outcomes reported in trials in coronavirus disease 2019 are extremely heterogeneous and of uncertain patient relevance, limiting their applicability for clinical decision-making. The aim of this workshop was to establish a core outcomes set for trials in people with suspected or confirmed coronavirus disease 2019.

**Design:**

Four international online multistakeholder consensus workshops were convened to discuss proposed core outcomes for trials in people with suspected or confirmed coronavirus disease 2019, informed by a survey involving 9,289 respondents from 111 countries. The transcripts were analyzed thematically. The workshop recommendations were used to finalize the core outcomes set.

**Setting:**

International.

**Subjects:**

Adults 18 years old and over with confirmed or suspected coronavirus disease 2019, their family members, members of the general public and health professionals (including clinicians, policy makers, regulators, funders, researchers).

**Interventions:**

None.

**Measurements:**

None.

**Main Results:**

Six themes were identified. “Responding to the critical and acute health crisis” reflected the immediate focus on saving lives and preventing life-threatening complications that underpinned the high prioritization of mortality, respiratory failure, and multiple organ failure. “Capturing different settings of care” highlighted the need to minimize the burden on hospitals and to acknowledge outcomes in community settings. “Encompassing the full trajectory and severity of disease” was addressing longer term impacts and the full spectrum of illness (e.g. shortness of breath and recovery). “Distinguishing overlap, correlation and collinearity” meant recognizing that symptoms such as shortness of breath had distinct value and minimizing overlap (e.g. lung function and pneumonia were on the continuum toward respiratory failure). “Recognizing adverse events” refers to the potential harms of new and evolving interventions. “Being cognizant of family and psychosocial wellbeing” reflected the pervasive impacts of coronavirus disease 2019.

**Conclusions:**

Mortality, respiratory failure, multiple organ failure, shortness of breath, and recovery are critically important outcomes to be consistently reported in coronavirus disease 2019 trials.

Coronavirus disease 2019 (COVID-19) was declared a global pandemic by the World Health Organization (WHO) on March 11, 2020. Patients with COVID-19 have an increased risk of mortality and multiple organ failure and debilitating symptoms including dyspnea, chest pain, and fatigue (1–6). The pandemic has imposed an unprecedented burden on healthcare systems worldwide, with demand for critical care exceeding capacity in some countries (7–10). As yet, there are no treatments proven to be effective (11).

In response to this crisis, clinical trials in COVID-19 have been initiated very rapidly. As of July 6, 2020, 4,098 trials were registered in the WHO International Clinical Trials Registry (12). The use of evidence from these trials to inform clinical decision-making is problematic, in part because the heterogeneity of outcomes reported across trials precludes robust comparisons across trials. Also, the outcomes of relevance to patients and clinicians may not always be reported. In a WHO review of outcomes used in 84 registered trials in COVID-19, six trials (7%) included mortality and 25 (30%) included lung injury indicated by oxygen saturation, respiratory failure, chest imaging, and oxygenation index (13).

The problems with heterogenous reporting of outcomes in trials are well known. Core outcome sets have been established to ensure that critically important outcomes are consistently reported in all trials (14, 15). There have been three initiatives to identify core outcomes for trials in COVID-19, all of which have established mortality and respiratory failure as core outcomes (13, 16–18). Mortality and respiratory failure in hospitalized patients were identified by all three initiatives (19). However, the prior initiatives involved a limited number of stakeholders (~70 to 135) and countries (1–25) (13, 16, 18, 19). Only one initiative involved patients and the public, who were all from China. This is of concern as patient-important outcomes are often omitted from trials (20).

To ensure broad inclusion of stakeholders globally, including patients and the general public, the COVID-19-Core Outcomes Set (COS) project was launched in March 2020 to establish a core outcomes set for trials in people with confirmed or suspected COVID-19 (21) across the full spectrum of disease and in all settings. The process was based on the Core Outcome Measures in Effectiveness Trials (COMET) framework (14) and involved a systematic review of outcomes reported in published and registered trials, an international online survey conducted in five languages involving 9,289 respondents from 111 countries (22) and four consensus workshops. In this report, we summarize the workshop discussions on establishing the core outcomes set and present the final COVID-19-COS core outcomes set.

## METHODS

### Overview and Context

Four online COVID-19-COS consensus workshops were convened from 14 to 15 of April 2020 using the video conferencing platform, Zoom, to discuss a proposed set of core outcomes that were identified from a prior international survey that involved 9,289 respondents (people with suspected or confirmed COVID-19 and their family members [*n* = 776]), members of the general public (*n* = 3,631), and health professionals (*n* = 4,882) from 111 countries (22, 23). The survey was conducted in five languages. At the workshop, we presented the top 10 rated outcomes identified by the survey respondents: mortality, respiratory failure, pneumonia, organ failure, lung function, lung scarring (fibrosis), sepsis/septic shock, shortness of breath, oxygen saturation, and hospitalization. These outcomes had a mean score greater than 7.5 (on a nine-point Likert scale, 7–9 being of critical importance), median greater than or equal to 8, with greater than 70% of respondents rating the outcome from 7 to 9 in each of the three stakeholder groups (patients/family members, public, and health professionals). The survey will be published separately.

### Participants and Contributors

We invited people with suspected or confirmed COVID-19 18 years old and over, family members, members of the general public, and health professionals (physicians with expertise in critical care medicine, pulmonary and respiratory disease, infectious disease, emergency medicine, cardiology, nephrology, nurses, multidisciplinary clinicians, researchers, funders, and policy makers) through the Steering Committee and Investigators and social media, with invitations sent by e-mail. In total, 95 attendees (including 17 with suspected/confirmed COVID-19) from 21 countries (Australia, Austria, Belgium, Brazil, Canada, Chile, China [mainland China and Hong Kong SAR], France, Germany, Italy, Japan, Portugal, Republic of Ireland, Saudi Arabia, South Africa, South Korea, Spain, Switzerland, United States, United Kingdom) participated. The full list of workshop attendees and investigators is provided in the acknowledgments.

### Workshop Program and Process

**Figure 1.**
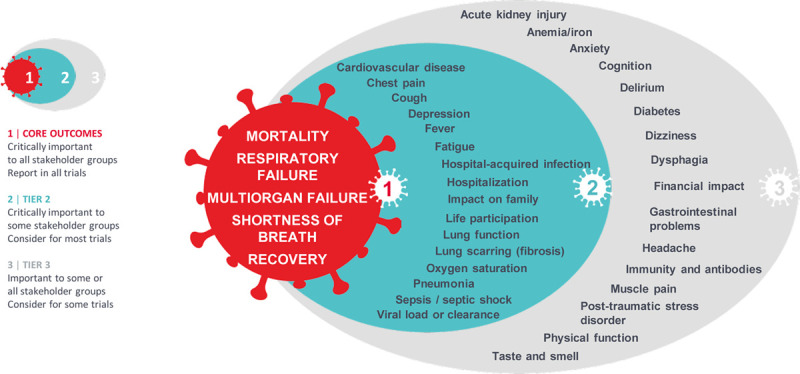
COVID-19-Core Outcomes Set (COS).

During each workshop, we presented the COVID-19-COS process, results from the survey, and a proposed core outcomes set. The attendees were then allocated into two virtual breakout rooms, each including people with COVID-19, members of the general public, and health professionals. The facilitator asked participants to discuss the proposed core outcomes. For feasibility of implementation, it was recommended that the core set should be comprised of three to five outcome domains, including at least one patient-reported outcome (14, 24). All 38 outcomes in the survey, including those added by survey respondents were shown to the participants. The five outcomes proposed for the core outcomes set presented at the workshop were as follows: mortality, respiratory failure, multiple organ failure, sepsis, and shortness of breath. Lung function, lung fibrosis, and pneumonia that were rated highly in the survey were not included in the proposed core outcomes set because of the overlap with respiratory failure and shortness of breath. All discussions were recorded and transcribed verbatim. The transcripts were imported into HyperResearch (ResearchWare Inc, version 3.0, Randolph, MA) for analysis. Using thematic analysis (25), author (A.T.) inductively identified themes on establishing core outcomes for trials in people with confirmed or suspected COVID-19. All attendees and investigators received the draft report, which included the final recommended COVID-19-COS core outcomes set (**Fig. 1**), and were invited to provide feedback by e-mail.

## RESULTS

The themes are described in the following section with selected quotations from the workshops provided in **Table 1**. **Box 1** outlines the recommendations from the consensus workshops.

**Box 1. Summary of the Workshop Recommendations to Consider in Establishing Core Outcome Domains for Trials in People With Suspected or Confirmed Coronavirus Disease 2019**

**Core outcome domains for trials in people with suspected or confirmed coronavirus disease 2019 should:**

Reflect the immediate goals of saving lives and minimizing the burden on the health system (i.e. mortality, respiratory failure, multiple organ failure).
Be applicable to care in a range of settings including hospital and community.
Be relevant and feasible to use in low-resource settings.
Inform decisions to manage both acute and long-term outcomes.
Capture long-term prognostic concerns (e.g. shortness of breath).
Include long-term functional outcomes that reflect the full disease trajectory including long-term outcomes (e.g. recovery).
Consider applicability to people with severe and mild-to-moderate COVID-19 (e.g. shortness of breath).
Include prevalent and debilitating symptoms (i.e. shortness of breath) that are meaningful and critically important to patients and as a prognostic indicator.
Include outcome domains that are adequately distinct (i.e. exclude sepsis from the core outcome set as multiple organ failure arising from COVID-19 infection is sepsis; lung health captures respiratory failure, lung function, lung scarring, pneumonia).

COVID-19 = coronavirus disease 2019.

**TABLE 1.** Selected Quotations

Themes	Quotations
Responding to the critical and acute health crisis	
Focus on saving lives	Right now the focus is keeping patients alive.
	There is one outcome that is far and away driving action and concern about this, and that is mortality. Everything else is secondary to the risk of death, for a self-limited acute disease where there is a substantial mortality. That is the major thing that we are trying to avert here, from the point of view of interventions. We are looking for interventions that will stop patients from dying. I would place mortality as out and away as a separate order of magnitude of importance.
	Mortality of course, because we see people dying, even young people without any comorbidity, they can end up on the ventilator and eventually die later on. We have several cases of young people, a very unfortunate one, a young girl of 12 yr who died in the ambulance to the hospital.
Preventing life-threatening complications	Right now the focus is keeping patients out of hospital, keeping them organ failure free out of the ICU and discharge home.
	Focus on the most severe end of the disease, these are problems that are going to, particularly mortality, multiple organ failure, sepsis, are clearly issues really for people who end up either in intensive care or who would go to intensive care were that to be an appropriate place for them to go.
	Multiple organ failure and the sepsis is a big one because I am one of the immunocompromised, I am in that high-risk category of COVID.
	Respiratory failure, multiple organ failure, sepsis are on the pathway to death unfortunately, and their significance is that that is what they lead to. Multiple organ failure is also important in my head because I do not know if he was going to then go into AKI because of COVID.
	It is very important respiratory failure because that is what everybody is so much afraid about to end up at the ICU and on the ventilator.
	It should be “severe” respiratory failure.
Capturing different settings of care	
Minimizing burden on hospitals	Respiratory failure this position of the patients whether they are going to be cared for on the general ward, or whether they need admission into intensive care.
	There is the need to have outcomes that capture the hospital resources. Outcomes such as admission to hospital, admission to ICU, need for a ventilator to capture the different terms and labels of resources that we have in decent healthcare systems.
	We are trying to protect the healthcare system.
	The most relevant thing here is actually the need that patients will require mechanical ventilation, which can be actually one of the important core outcomes in patients. We have two separate basically two groups of patients with COVID. The patients who are treated in a normal ward. They are critical to certain extent until they need an ICU admission, and once the patients are admitted to the ICU, you will have a different situation actually with other outcomes of interests.
Recognizing events occurring in community contexts	We have 258 cases who died and 134 died outside of the hospital, mostly in aged care facilities. We were facing a bottleneck with the ability to admit patients and ventilate patients in ICU. A lot of people in aged care facility who were severely ill were not even admitted in hospital, because we knew that if they were admitted they would have required ICU which we could not offer to them.
	People who have suspected COVID-19 have the milder conditions would not necessarily end up at hospitals.
	I was in the hospital briefly, but not hospitalized per se. Multiple organ failure and sepsis were not an issue for me, but the shortness of breath, the breathing issues, I had a significant headache that I cannot begin to describe the severity of it.
	How much of these outcomes will be used in community studies as opposed to in hospital studies? My understanding is that COVID, certainly in the United Kingdom is absolutely prevalent in the community and we are not being tested so we do not know.
	I was at home for the duration of my illness. Luckily, I did not have it to a point where I needed to go to hospital or anything. My main thing was that I was so exhausted I could not move or do anything.
Ensuring relevance and feasibility in low resource regions	The options of ventilation and so on are restrictive. It is very relevant to realize that round about 87% of the world’s population actually live in low middle-income class countries. It literally has been a day and night job in our setting and for many of our friends elsewhere, and in fact on the continent of Africa… some of whom may not be able to in any context even offer ventilation—it is just not feasible.
	We are several weeks behind most of you, so launching the planning phases but we are seeing increasing numbers of patients and where ICU facilities do exist, and we are getting away in many instances the use of polymasks and proning a patient, without necessarily invasively ventilating them and patients who got saturation’s of 70% and 80%, where ordinarily in any other context these patients would have been invasively ventilated.
	The other thing that is really important is that we make sure that whatever definition we use is applicable across as wide a range of context as possible. So ECMO and something is very important in the developed world and so forth, but there are countries where ECMO is simply not available. In fact there are countries where even mechanical ventilation is not available. So we must make sure I think that this is as applicable across contexts as we can manage whilst not collapsing things of saying advanced respiratory support is all the same, but also to try and capture from as many contexts as possible so that trials do take place in less well resourced areas can also take part in the core outcome set.
	I am speaking on behalf of the low- and middle-income countries. We have seen a lot of patients with multiple organ failure because these patients arrived late in the hospital because sometimes they are stuck in the other parts of the national healthcare system. Multiple organ failure will be an important issue in this scenario.
Encompassing the full trajectory and severity of disease	
Addressing prognostic uncertainty and long-term concerns	The most important thing to me would not be if I died with COVID, it is just if I got it and was worse and it destroyed say half my lung function or to quality of life.
	It is about ability to function afterwards. I have no comorbidities to my knowledge, but what is worrying me now is post viral fatigue and recovery, because it is been a very long time since I’ve spent 2 wk in bed, and I am showing no signs of being strong enough to even function in the house, never mind go back to work. What is concerning me about a long-term recovery is that kind of post viral thing, and then also are there lung injuries for people that do not even have comorbidities? Am I at risk of suffering some term loss of some lung function?
	I am at day 26 now of having the disease, and I still have a fever, and I still have a really high heart rate, and I still have shortness of breath and chest pain and a cough. I am grateful that I am not in hospital with more dire symptoms. I am a musician and I use my lungs for a lot of things. The long-term effects of the disease would be something good to look at.
	In terms of fatigue, I am a very active person and I cannot stand for more than 10 min anymore, and that is a really weird feeling in my 20s. I am really scared when they do tell me that I can go and get an x-ray, what my lungs are going to show because I have never had that kind of chest pain and shortness of breath before.
	I had side respiratory failure and I would like to make a pitch for some long-term consequence of COVID-19 infection being included in the core outcome, such as pulmonary fibrosis. Because you could imagine a kind of discreet choice experiment, where a clinician or a patient or a health provider has to decide, “Do we ventilate harder to get the patient off the ventilator sooner, but risk more pulmonary fibrosis long-term?”
Applicable to mild and moderate disease	I treated about 30 patients with COVID-19, some mild patient, some mild disease. They were hospitalized on a standard floor, so they were treated with first some oxygen by the nasal prongs.
	There will be a period of recovery. This week I can have a shower and go downstairs and have breakfast and I am not short of breath, whereas last week I could not do that. So that is an indicator of some measure of recovery.
	Shortness of breath can be a good outcome parameter in the sub pool of patients who are admitted to normal ward, not patients in ICU.
	Fatigue, shortness of breath are very important from patient perspective especially in patients who have milder form of disease.
	Absence of disease [recovery], which surely is one of the outcomes that one is trying to accomplish when one is conducting a trial is to cure people and to get rid of the disease.
Distinguishing overlap, correlation, and collinearity	
Symptoms having distinct value	Shortness of breath was one that was quite debilitating for me, in terms of trying to do some normal things like washing, like cloth washing or tidying the flat, I had get very short of breath. It was the shortness of breath and the fatigue that lasted the longest, and it was at that stage when I did not have the fever or anything else. How long until these symptoms go so I can get back to a level of normality in life, and go back to work, do gentle exercise?
	The issue with shortness of breath is, it is not very specific but that may be a good thing, because it is more sensitive in that regard.
	Shortness of breath is strongly related to ICU-acquired weakness.
	We should be including shortness of breath to capture that patient experience.
	I consider myself somebody who has got a moderate case. I have been dealing with this almost 4 wk now and I am still having shortness of breath and other symptoms. It is troublesome not to be able to breathe.
	Even though I do not have the virus in my body, I am still having shortness of breath, chest tightness, and I am still coughing as well.
	Shortness of breath maybe important in self isolated patients at home, usually a chest radiograph cannot detect the pneumonia in COVID-19. Some of these patients who have shortness of breath, can, progress quickly to the respiratory failure, so the shortness of breath maybe can be the only sign of the respiratory failure or pneumonia.
	There are ICU data showing that shortness of breath, even a patient on a ventilator has some prognostic significance. So shortness of breath seems to be different from respiratory failure and [the patient’s] comments about her father having respiratory failure but not being short of breath, quite pertinent. I think it matters to the person about how they feel. And so I would favor including shortness of breath.
Clarifying causal pathways	There is a major overlap between multi organ failure and sepsis, particularly with the definition change in sepsis, where organ failure is vital to that. Those are really the same thing because this is drive by an infection so therefore by definition any multi organ failure arising from COVID is sepsis.
	If we are going to combine them to delete the sepsis terminology. Sepsis is a very vague condition.
	Multiple organ failure is a bit tenuous but it is important, we have seen a lot of kidney damage, a lot of AKI, a lot of need for dialysis and it seems to be effecting outcomes or how well or how poorly they are going to do.
	I have been rounding for almost 4 wk now in a row, is very variable if the patients are going to go into multiple organ failure or not. The one that I have in the unit, obviously they have more chance of having multiple organ failure.
	Multiple organ failure and sepsis was somewhere on the causal pathway between the respiratory failure and mortality.
	Most of the cases that we saw so far in our institution, they died not only from the respiratory failure, they died actually from non-respiratory organ failure. So non-respiratory organ failure can also be an important outcome parameter in these patients, mostly coagulation failure.
Recognizing adverse events	
	There is a lot of studies that are being started that are evaluating hydroxychloroquine for example, which has adverse events. So especially the population of patients who are not as severe, so instituting this treatment can be problematic and can institute adverse events.
	Adverse events are often intervention specific and so they are not going to have a common domain across all different drug classes or other intervention foci in an area here where there does not seem to be any compelling cases or effective interventions at present, that just adds to the uncertainty.
	A topic that is of relevance is also the harms, the side effects. We are potentially going to be trialling a lot of novel interventions. The question is, what is the trade-off between the potential benefits, the respiratory failure, whatever, and those harms which probably we are going to have to accept to obtain certain benefits.
	We should be aware of the variation in approach to treatment around the world and the way that that is evolving quite rapidly. Outcomes that are more based on physiologic state rather than the initiation of a particular therapeutic intervention may be a better approach.
	Being cognizant of family and psychosocial wellbeing	
	The impact on family is quite huge to have those social issues to be looked at as well, considering when we are talking about people isolated in hospitals and do not have any contact with their family and how difficult that is and how stressful that is and could actually equate to you actually being sicker and deteriorating a lot quickly into depression and anxiety. We have seen people over on social media where they have had to stand outside and speak to their loved one on a walkie talkie to say goodbye because they are dying.
	It is still depression to me or psychologic issues associated with the disease is still a very big thing, both for the family member. I was actually out of hospital before my father was, and I gave it to my father. I did not fall into depression, but I can actually see how people will have a lot of anxiety about passing it to their family members and also that they may go into depression.
	Are we picking up anything on psychologic wellbeing or worry, anxiety, sense of control.
	And then the last thing specific to the safety net population is this idea or this outcome of vitality and financial stability. I had several patients that actually left against medical advice, so still requiring oxygen and hospitalization, but left because they thought they needed to go back to work and there was nothing our hospital could do to keep them there in the hospital or to let their employer know that they were infected, but they had to go back to work because they were day laborers and needed to make an income for their family.
	I was worried about whether I had given it to anybody when I went to the doctors. I was just constantly worried and I do not feel like anybody’s talking about the anxiety side of what having coronavirus does.

AKI = acute kidney injury, COVID = coronavirus disease, ECMO = extracorporeal membrane oxygenation.

### Responding to the Critical and Acute Health Crisis

#### Focus on Saving Lives.

Mortality was consistently identified as the most important outcome, which reflected the pressing primary goal of “keeping patients alive” and managing the “crisis” of the global pandemic. Death was identified as the worst outcome and was “far and away driving action and concern about [COVID-19], the major thing we’re trying to avert from the point of view of interventions.”

#### Preventing Life-Threatening Complications.

The high priority placed on mortality, respiratory failure, and multiple organ failure resonated with the urgent need to manage severe and critical cases of COVID-19. It was suggested that respiratory failure should be defined as “‘severe’ respiratory failure because that’s what everybody is so much afraid of, to end up at the ICU and on the ventilator.” Some patients were concerned that many who were placed on a ventilator did not recover. These severe outcomes were particularly feared among vulnerable populations—“multiorgan failure is a big one because I’m one of the immunocompromised; I’m in that high-risk category of COVID having severe reactions.”

### Capturing Different Settings of Care

#### Minimizing Burden on Hospitals.

The need to “protect the healthcare system” supported the inclusion of outcomes relevant to the hospital setting. Some countries encountered difficulties in mobilizing healthcare resources during the early phase of the pandemic. Respiratory failure was important because it determined if patients would require admission to intensive care. Health professionals suggested that the use of hospital resources should be embedded in how the core outcomes were defined—“kidney failure could be defined as need for kidney replacement therapy. Respiratory failure could be defined based on what respiratory support you require.”

#### Recognizing Events Occurring in Community Contexts.

Many people with COVID-19 were managed at home or in the community (e.g. aged care facilities), thus including outcomes relevant outside of the hospital setting was warranted. Patients who were not hospitalized emphasized that severe symptoms, including shortness of breath, fatigue, and headache, as well as recovery, were important—“there might be a distinction between people who are hospitalized and critically ill versus people who are managing it at home and recovering.” In some countries, people could not be admitted to ICU owing to limited hospital capacity; therefore, some participants advised that COVID-19 related mortality “needed to include out-of-hospital deaths.”

#### Ensuring Relevance and Feasibility in Low-Resource Regions.

In some settings, particularly in low-income countries, mechanical ventilation, and extracorporeal membrane oxygenation were not available—“we are getting away in many instances with proning a patient [positioning a patient flat on the stomach with the chest and head facing down], without necessarily invasively ventilating them; these are patients who have got saturations of 70 and 80%, where ordinarily in any other context those patients would have been invasively ventilated.” Therefore, the definition and measure of respiratory failure also needs to also be applicable in low-income countries. Multiple organ failure was highly relevant in low- and middle-income countries—“we have seen a lot of patients with multiple organ failure because these patients arrived late in the hospital, because sometimes they are stuck in the other parts of the healthcare system [when they should be in ICU].”

### Encompassing the Full Trajectory and Severity of Disease

#### Addressing Prognostic Uncertainty and Long-Term Concerns.

The long-term impacts of COVID-19 are relevant. However, the specific outcomes of most importance were uncertain because of the lack of data on longer-term outcomes. Patients wanted to know about time to recovery—“what is worrying me now is post viral fatigue and recovery, because it’s been a very long time since I’ve spent two weeks in bed, and I’m showing no signs of being strong enough to even function in the house, never mind go back to work. That’s what’s concerning me about a long-term recovery is that kind of post viral thing.” Health professionals suggested that recovery should be a core outcome—“I was surprised to not see recovery as a core outcome in the first category.” Participants suggested consideration of ongoing symptoms such as shortness of breath, fatigue, chest pain, and cough—“I’m in day 35 and I’m still struggling with shortness of breath.” Some patients were concerned about long-term impacts on lung health (e.g. lung function, lung scarring)—“I am really scared when they do tell me that I can go and get an x-ray, what my lungs are going to show because I’ve never had that kind of chest pain and shortness of breath before.” Long-term outcomes were relevant to make trade-offs in decision-making—“imagine a kind of discrete choice experiment to decide, ‘do we ventilate harder to get the patient off the ventilator sooner, but risk more pulmonary fibrosis long-term?’ There’s a balance between the acute outcomes and the long-term consequences.”

#### Applicable to Mild and Moderate Disease.

Shortness of breath, oxygen saturation, pneumonia, recovery, and fatigue were suggested to be important for people who did not have severe COVID-19—“if I were asymptomatic and in a trial I certainly would be interested in shortness of breath, but I’d also be interested in whether I needed to get admitted to the hospital.” To cover the spectrum of disease severity, participants recommended that outcomes related to postacute illness recovery be included—“absence of disease [recovery], which surely is one of the outcomes that one is trying to accomplish when one is conducting a trial is to cure people and to get rid of the disease.” Patients referred to recovery as being able to do usual activities—“There will be a period of recovery. This week I can have a shower and go downstairs and have breakfast and I’m not short of breath, whereas last week I couldn’t do that. So that’s an indicator of some measure of recovery.”

### Distinguishing Overlap, Correlation, and Collinearity

#### Symptoms Having Distinct Value.

Shortness of breath was a symptom that was “debilitating,” “lasted the longest,” and prevented the ability to do usual daily tasks and thus captured an important aspect of the patient experience of COVID-19. It was recognized that shortness of breath could have value as a prognostic indicator because “a chest radiograph may not always detect pneumonia in COVID-19 and some of these patients who have shortness of breath can progress quickly to respiratory failure, so it may be the only sign of respiratory failure or pneumonia.” Health professionals advised that shortness of breath could be a symptom of COVID-19 and also related to ICU-acquired diaphragmatic weakness.

#### Clarifying Causal Pathways.

Health professionals noted that sepsis was included in the definition of multiple organ failure—“sepsis is defined as organ failure due to infection. All the patients have infection, by definition.” Respiratory failure could “dominate the category of multiple organ failure.” However, some argued that multiple organ failure was justifiable as a core outcome because kidney damage or cardiovascular disease had been observed in people with COVID-19. Lung outcomes (respiratory failure, lung function, lung scarring, and pneumonia) were all of high priority, however were on a continuum inclusive of respiratory failure.

### Recognizing Adverse Events

It was expected that “we are going to be trialling a lot of novel interventions” and participants urged awareness of the “variation in approach to treatment around the world and the way that that’s evolving quite rapidly.” However, without compelling cases for effective treatments at present and with little commonality across different interventions (e.g. drug classes), potential intervention-specific adverse events may be excluded from the core outcomes set.

### Being Cognizant of Family and Psychosocial Wellbeing

Due to the potentially severe nature of COVID-19 and requirement for quarantine, emphasis was placed on the profound impacts on the psychosocial wellbeing of patients and their families. Being in isolation was thought to exacerbate sickness, deterioration, depression, and anxiety for patients and their families—“we’ve seen people where they’ve had to stand outside and speak to their loved one on a walkie talkie to say goodbye because they’re dying.” Patients experienced guilt, fear, and depression related to infecting others.

### The COVID-19-COS Core Outcomes Set

The recommendations (Box 1) arising from the workshop were used to finalize the core outcomes set based on review by the Steering Committee and investigators. Sepsis was moved from the initial core outcomes set into Tier 2 because of overlap with multiple organ failure. Lung function, lung fibrosis, and pneumonia remained in Tier 2 to avoid overlap with respiratory failure and shortness of breath. Shortness of breath was retained in the core outcomes set because it was the most important patient-reported outcome and was a symptom relevant across the spectrum of COVID-19 and across the full trajectory of an individual’s disease course. It was strongly and consistently recommended that a longer term outcome should be included. However, the top 10 ranked outcomes from the survey comprised only of acute and severe outcomes. Recovery (defined in the survey as how long it takes to recover, i.e., feel better, no longer having symptoms) was the highest rated long-term outcome and was moved from Tier 2 into the core outcomes set as in response. Six outcomes relating to symptoms and psychosocial and family impact (based on those that were highest ranked in the survey—chest pain, cough, depression, fatigue, impact on family, life participation) were moved from Tier 3 to Tier 2. The final COVID-19-COS core outcome domains are shown in Figure 1.

## DISCUSSION

Overall, people with suspected or confirmed COVID-19, family members, the public, and health professionals agreed that mortality, respiratory failure, and multiple organ failure were the most critically important outcomes that should be core outcomes for all trials in COVID-19. These outcomes reflected the immediate goals of saving lives, preventing life-threatening complications, and protecting the healthcare system that were paramount in the current pandemic. Shortness of breath was identified as a persistent and debilitating symptom that impaired the ability to perform daily activities, was relevant to all levels of disease severity, and provided meaningful information about the patient experience of COVID-19. This symptom was also seen to have potential value as a diagnostic and prognostic indicator for lung health including pneumonia and respiratory failure or as a long-term outcome, for example, a sign of ICU-acquired weakness. All stakeholders emphasized the importance of capturing the full severity and trajectory of disease in the core outcomes set, including long-term outcomes. Therefore, recovery was included in the core outcomes set.

The workshop discussions and recommendations informed the selection of the core outcome domains to be reported in trials in people with confirmed or suspected COVID-19: mortality, respiratory failure, multiple organ failure, shortness of breath, and recovery (Fig. 1, Panel 1).

Mortality, respiratory failure, multiple organ failure, and recovery have been identified as core outcomes for COVID-19 by recent initiatives (13, 16, 17). The WHO core outcomes set for clinical research included three domains: survival (all-cause mortality at hospital discharge or 60 d), viral burden, and clinical progression (including need for interventions for respiratory and multiple organ failure) (13). The COS-COVID core outcomes set included viral load, hospitalization, oxygen saturation, respiratory failure (duration of mechanical ventilation), and mortality (16). Qiu et al (17) identified eight outcome domains, including recovery time and mortality, respiratory outcomes, and non-specified symptoms. The COVID-19-COS core outcomes set includes the patient-reported outcome of shortness of breath, which has not been previously identified. Shortness of breath is a common and distressing symptom in people with lung disease and a potential predictor of mortality (26). A study in pulmonary rehabilitation in patients with chronic obstructive pulmonary disease identified shortness of breath as one of the most important outcomes for patients, caregivers, and health professionals (27). An international Delphi survey involving researchers, clinicians, patients/caregivers, and funders identified pulmonary symptoms as a core domain for survivors of acute respiratory failure after discharge from the hospital (28).

Further work is needed to identify valid core outcome measures for the core outcome domains that can be feasibly implemented in all trials in people with suspected or confirmed COVID-19. This will involve the review of established definitions and measures (including core measures) for mortality, respiratory failure, multiple organ failure, and recovery and possible pilot and validation work to establish a patient-reported outcome measure for shortness of breath that is psychometrically robust and of minimal burden to implement.

## CONCLUSIONS

With the rapidly growing body of evidence from clinical trials, urgent implementation of core outcomes in trials in COVID-19 can help to improve the consistency of reporting outcomes that are critically important to patients, family members, the public, and health professionals. This can better inform decision-making in the context of this pandemic and strengthen the value of trials to inform the management of people with suspected and confirmed COVID-19 and hopefully improve patient outcomes.

## ACKNOWLEDGMENTS

We acknowledge, with permission, all the patients, family members, members of the public and health professionals who attended the consensus workshops: Workshop 1: Paolo Ferrari, Ella Flemyng, Yen Ho, Lynn Laidlaw, Lowell Ling, Anne McKenzie, Mervyn Mer, Saad Nseir, Margaret O’Hara, Sam Petty, Simone Piva, Pedro Povoa, Karen Rawden, Tessa Richards, Regina Rodriguez-Martin, Alan Smyth, Jorgen Vestbo, Junhua Zhang, Jonathan Craig, Amanda Baumgart, Tess Cooper, Elyssa Hannan, Karine Manera, Armando Teixeira-Pinto, Andrea Viecelli, and Allison Tong; Workshop 2: Justin Denholm, Davina Ghersi, Tamara Johnson, Deb Kilroy, Eduardo Lorca, Karen Maschke, Sangeeta Mehta, Andrew Conway Morris, Loisann Openshaw, Tara Parker, Mark Reid, Ian Saldanha, Nicole Scholes-Robertson, Ning Shen, Tom Snelling, Alexis Turgeon, Tari Turner, Denise Warzel, Dave White, Laila Woc-Colburn, Jonathan Craig, Armando Teixeira-Pinto, Martin Howell, Andrea Viecelli, Andrea Matus-Gonzalez, Patrizia Natale, Amanda Baumgart, and Allison Tong; Workshop 3: Neill Adhikari, Samaya Anumudu, Jairo Barrantes Perez, Fernando Bozza, Elaine Chan, Derek Chew, Amanda Dominello, Ivor Douglas, Charles Gomersall, David Henry, Khaled Kerouani, Yong-Lim Kim, Jaehee Lee, Jonathan Levesque, Deena Lynch, Guy Marks, Belinda Ng, Sallie Pearson, Helen Reddel, Giovanni Strippoli, Jo Watson, Jonathan Craig, Amanda Baumgart, Amelie Bernier-Jean, Nicole Evangelidis, Elyssa Hannan, Emma Liu, Armando Teixeira-Pinto, Andrea Viecelli, and Allison Tong; Workshop 4: Kimberley Ambrose, Yaseen Arabi, Luciano Azevedo, Lilia Cervantes, Sally Crowe, Chris Douglas, Julian Elliott, David Malins, John Marshall, Brian McGuire, Yasser Sakr, Naoki Shimizu, Thomas Staudinger, Antoni Torres, Wim Van Biesen, Angela Wang, Paula Williamson, Timo Wolf, Germaine Wong, Amanda Baumgart, Yeoungjee Cho, Elyssa Hannan, Charlie McLeod, Valeria Saglimbene, Armando Teixeira-Pinto, Andrea Viecelli, and Allison Tong.

## COVID-19-Core Outcomes Set (COS) Workshop Investigators (Attending and Nonattending Contributors) for Group Authorship

**Table:**

Neill K. J.	Adhikari	Sunnybrook Health Sciences Centre and University of Toronto	Toronto	Canada
Kimberley	Ambrose	Consumer—patient	Cambridge	United Kingdom
Samaya	Anumudu	Baylor College of Medicine	Houston	United States
Yaseen	Arabi	King Saud Bin Abdulaziz University for Health Sciences	Riyadh	Saudi Arabia
Ximena	Atilano-Carsi	National Institute of Health Sciences and Nutrition	Mexico City	Mexico
Luciano	Azevedo	Hospital Sirio-Libanes and University of Sao Paulo	São Paulo	Brazil
Jairo	Barrantes Perez	Baylor College of Medicine	Houston	United States
Amanda	Baumgart	The University of Sydney	Sydney	Australia
Amelie	Bernier-Jean	The University of Sydney	Sydney	Australia
Andrew	Bersten	Flinders University	Adelaide	Australia
Fernando	Bozza	National Institute of Infectious Disease	Rio de Janero	Brazil
Simon	Carter	The University of Sydney	Sydney	Australia
Sebastian	Cebrera	The University of Chile	Santiago	Chile
Lilia	Cervantes	Denver Health	Denver	United States
Elaine	Chan	Westmead Hospital	Sydney	Australia
Derek	Chew	Flinders University	Adelaide	Australia
Yeoungjee	Cho	University of Queensland	Brisbane	Australia
Deborah	Cook	McMaster University	Hamilton	Canada
Tess	Cooper	The University of Sydney	Sydney	Australia
Jonathan	Craig	Flinders University	Adelaide	Australia
Sally	Crowe	Crowe Associates Ltd	Oxon	United Kingdom
Justin	Denholm	University of Melbourne	Melbourne	Australia
Amanda	Dominello	Consumer—public	Sydney	Australia
Chris	Douglas	Consumer—patient	Perth	Australia
Ivor	Douglas	Denver Health	Denver	United States
Carolina	Echegoyen	Centro de Salud Familiar Santa Amalia	Santiago	Chile
Julian	Elliott	Monash University	Melbourne	Australia
Nicole	Evangelidis	The University of Sydney	Sydney	Australia
Paolo	Ferrari	Ente Ospedaliero Cantonale	Bellinzona	Switzerland
Ella	Flemyng	Cochrane	London	United Kingdom
Davina	Ghersi	National Health and Medical Research Council	Canberra	Australia
Charles	Gomersall	The Chinese University of Hong Kong	Hong Kong	China
Sally	Green	Monash University	Melbourne	Australia
Chandana	Guha	The University of Sydney	Sydney	Australia
Elyssa	Hannan	The University of Sydney	Sydney	Australia
Camilla	Hanson	The University of Sydney	Sydney	Australia
Tess	Harris	PKD International	London	United Kingdom
David	Henry	Bond University	Gold coast	Australia
Sophie	Hill	La Trobe University	Melbourne	Australia
Yen	Ho	Consumer—public	Sydney	Australia
Peter	Horby	University of Oxford	Oxford	United Kingdom
Martin	Howell	The University of Sydney	Sydney	Australia
Ivan	Hung	The University of Hong Kong	Hong Kong	China
Tamara	Johnson	Consumer—patient	Indiana	United States
Angela	Ju	The University of Sydney	Sydney	Australia
Ayano	Kelly	The Children’s Hospital at Westmead	Sydney	Australia
Khaled	Kerouani	Consumer—patient	Boston	United States
Rabia	Khalid	The University of Sydney	Sydney	Australia
Deb	Kilroy	Consumer—patient	Brisbane	Australia
Yong-Lim	Kim	Kyungpook National University	Daegu	South Korea
Katharina	Kohler	University of Cambridge	Cambridge	United Kingdom
Lynn	Laidlaw	Consumer—public	London	United Kingdom
Jaehee	Lee	Kyungpook National University	Daegu	South Korea
Jonathan	Levesque	University of Montreal	Montreal	Canada
Lowell	Ling	Chinese University of Hong Kong	Hong Kong	Hong Kong
Emma	Liu	The Children’s Hospital at Westmead	Sydney	Australia
Eduardo	Lorca	University of Chile	Santiago	Chile
Deena	Lynch	Consumer—patient	Brisbane	Australia
David	Malins	Consumer—community	London	United Kingdom
Karine	Manera	The University of Sydney	Sydney	Australia
Guy	Marks	University of New South Wales	Sydney	Australia
John	Marshall	University of Toronto	Toronto	Canada
Karen	Maschke	The Hastings Center	New York	United States
Andrea	Matus Gonzalez	The University of Sydney	Sydney	Australia
Brian	McGuire	National University of Ireland	Galway	Ireland
Anne	McKenzie	Telethon Kids Institute	Perth	Australia
Charlie	McLeod	Perth Children’s Hospital	Perth	Australia
Sangeeta	Mehta	University of Toronto	Toronto	Canada
Mervyn	Mer	University of Witwatersrand	Johannesburg	South Africa
Andrew Conway	Morris	University of Cambridge	Cambridge	United Kingdom
Patrizia	Natale	University of Bari	Bari	Italy
Belinda	Ng	Consumer—patient	Hong Kong	China
Saad	Nseir	Lille University	Lille	France
Margaret	O’Hara	Consumer—patient	Birmingham	United Kingdom
Loisann	Openshaw	Consumer—patient	Arizona	United States
Tara	Parker	Consumer—patient	Marietta Atlanta	United States
Sallie	Pearson	University of New South Wales	Sydney	Australia
Sam	Petty	University of Cambridge	Cambridge	United Kingdom
Simone	Piva	University of Brescia	Brescia	Italy
Gabriela	Pomiglio	Aterym SRL Cordoba Military Hospital	Cordoba	Argentina
Pedro	Povoa	Centro Hospitalar Lisboa Ocidental	Lisbon	Portugal
Karen	Rawden	Consumer—patient	Towcester	United Kingdom
Helen	Reddel	The University of Sydney	Sydney	Australia
Mark	Reid	Denver Health	Denver	United States
Tessa	Richards	British Medical Journal	London	United Kingdom
Regina	Rodriguez-Martin	Consumer—patient	Chicago	United States
Sarah	Rowan	Denver Health	Denver	United States
Valeria	Saglimbene	The University of Sydney	Sydney	Australia
Yasser	Sakr	Jena University Hospital	Jena	Germany
Ian	Saldanha	Brown University School of Public Health	Providence	United States
Benedicte	Sautenet	University of Tours	Tours	France
Nicole	Scholes-Robertson	The University of Sydney	Sydney	Australia
Ning	Shen	Peking University Third Hospital	Beijing	China
Jenny	Shen	University of California Los Angeles	Los Angeles	United States
Naoki	Shimizu	St. Marianna University School of Medicine	Kawasaki	Japan
Alan	Smyth	University of Nottingham	Nottingham	United Kingdom
Tom	Snelling	The University of Sydney	Sydney	Australia
Thomas	Staudinger	Medical University of Vienna	Vienna	Austria
Giovanni	Strippoli	University of Bari	Bari	Italy
Alix	Surber	Consumer—patient	Atlanta	United States
Anneliese	Synnot	La Trobe University	Melbourne	Australia
Armando	Teixeira-Pinto	The University of Sydney	Sydney	Australia
Allison	Tong	The University of Sydney	Sydney	Australia
Antoni	Torres	University of Barcelona	Barcelona	Spain
Alexis	Turgeon	Université Laval	Quebec	Canada
Tari	Turner	Monash University	Melbourne	Australia
Marcelo	Urra	University of Chile	Santiago	Chile
Wim	Van Biesen	University of Ghent	Ghent	Belgium
Jorgen	Vestbo	The University of Manchester	Manchester	United Kingdom
Andrea K.	Viecelli	University of Queensland	Brisbane	Australia
Angela	Wang	The University of Hong Kong	Hong Kong	China
Denise	Warzel	National Institutes for Health	Washington DC	United States
Jo	Watson	Department of Health	Canberra	Australia
Steve	Webb	Royal Perth Hospital	Perth	Australia
David	Wheeler	University College London	London	United Kingdom
Dave	White	Consumer—community	NY	United States
Paula	Williamson	University of Liverpool	Liverpool	United Kingdom
Laila	Woc-Colburn	Baylor College of Medicine	Houston	United States
Timo	Wolf	Goethe University	Frankfurt	Germany
Germaine	Wong	The University of Sydney	Sydney	Australia
Junhua	Zhang	Tianjin University of Traditional Chinese Medicine	Tianjin	China

## References

[1] Zhou F Yu T Du R Clinical course and risk factors for mortality of adult inpatients with COVID-19 in Wuhan, China: A retrospective cohort study. Lancet. 2020; 395:1054–1062 32171076 10.1016/S0140-6736(20)30566-3 PMC7270627

[2] Chen N Zhou M Dong X Epidemiological and clinical characteristics of 99 cases of 2019 novel coronavirus pneumonia in Wuhan, China: A descriptive study. Lancet. 2020; 395:507–513 32007143 10.1016/S0140-6736(20)30211-7 PMC7135076

[3] Bhatraju PK Ghassemieh BJ Nichols M COVID-19 in critically ill patients in the seattle region - case series. N Engl J Med. 2020; 382:2012–2022 32227758 10.1056/NEJMoa2004500 PMC7143164

[4] Grasselli G Zangrillo A Zanella A Baseline characteristics and outcomes of 1591 patients infected with SARS-CoV-2 admitted to ICUs of the Lombardy Region, Italy. J Am Med Assoc. 2020; 323:1574–1581 10.1001/jama.2020.5394 PMC7136855 32250385

[5] Chen R Liang W Jiang M Risk factors fo fatal outcome in hospitalized subjected with coronavirus disease 2019 from a nationwide analysis in China. Chest. 2020; 158:97–105 32304772 10.1016/j.chest.2020.04.010 PMC7158802

[6] Auld SC Caridi-Scheible M Blum JM ICU and ventilator mortality among critically ill adults with coronavirus disease 2019. Crit Care Med. 2020 5 26. [online ahead of print] 10.1097/CCM.0000000000004457 PMC7255393 32452888

[7] Lee CCM Thampi S Lewin B Battling COVID-19: Critical care and peri-operative healthcare resource management strategies in a tertiary academic medical centre in Singapore. Anaesthesia. 2020; 75:861–871 32267963 10.1111/anae.15074 PMC7262214

[8] Garcia-Castrillo L Petrino R Leach R European society for emergency medicine position paper on emergency medical systems’ response to COVID-19. Eur J Emerg Med. 2020; 27:174–177 32243317 10.1097/MEJ.0000000000000701 PMC7202106

[9] Ramachandran P Swamy L Kaul V A national strategy for venitlator and ICU resource allocation during the COVID-19 pandemic. Chest. 2020; S0012-3692:31398–2 10.1016/j.chest.2020.04.050 PMC7217111 32413343

[10] Maves RC Downar J Dichter JR Triage of scarce critical care resources in COVID-19 an implementation guide for regional allocation: An expert panel report of the task force for mass critical care and the American College of Chest Physicians. Chest. 2020; 158:212–225 32289312 10.1016/j.chest.2020.03.063 PMC7151463

[11] Sanders JM Monogue ML Jodlowski TZ Pharmacologic treatments for coronavirus disease 2019 (COVID-19): A review. JAMA. 323:1824–1836 10.1001/jama.2020.6019 32282022

[12] World Health Organization. International Clinical Trials Registry Platform (ICTRP). 2020 Geneva, Switzerland Available at: https://www.who.int/ictrp/en/. Accessed April 20, 2020

[13] WHO Working Group on the Clinical Characterisation and Management of COVID-19 infection: A minimal common outcome measure set for COVID-19 clinical research. Lancet Infect Dis. 2020; 20:e192–e197 32539990 10.1016/S1473-3099(20)30483-7 PMC7292605

[14] Williamson PR Altman DG Bagley H The COMET Handbook: Version 1.0. Trials. 2017; 18:280 28681707 10.1186/s13063-017-1978-4 PMC5499094

[15] Chalmers I Bracken MB Djulbegovic B How to increase value and reduce waste when research priorities are set. Lancet. 2014; 383:156–165 24411644 10.1016/S0140-6736(13)62229-1

[16] Jin X Pang B Zhang J Core outcome set for clinical trials on coronavirus disease 2019 (COS-COVID). Engineering. 2020 3 18 [online ahead of print] 10.1016/j.eng.2020.03.002 PMC7102592 32292626

[17] Qiu R Zhao C Liang T Core outcome set for clinical trials of COVID-19 based on traditional Chinese and Western medicine. Front Pharmacol. 2020; 11:781 32574235 10.3389/fphar.2020.00781 PMC7265660

[18] World Health Organization. A minimal common outcome measure set for COVID-19 clincial research. Lancet Infect Dis. 2020; 20:e192–e197 32539990 10.1016/S1473-3099(20)30483-7 PMC7292605

[19] COMET: The COMET Initiative: Core outcome Set Developers’ Response to COVID-19. 2020 Available at: http://www.comet-initiative.org/Studies/Details/1538. Accessed April 22, 2020

[20] Gandhi GY Murad MH Fujiyoshi A Patient-important outcomes in registered diabetes trials. JAMA. 2008; 299:2543–2549 18523223 10.1001/jama.299.21.2543

[21] Tong A It Takes a Community to Establish Core Outcomes for Research in COVID-19. 2020 The BMJ Opinion; Available at: https://blogs.bmj.com/bmj/2020/04/09/it-takes-a-community-to-establish-core-outcomes-for-research-in-covid-19/. Accessed April 22, 2020

[22] COVID-19-COS: COVID-19-COS Core Outcomes Set. 2020 Available at: http://www.covid-19-cos.org/cos/. Accessed July 1, 2020

[23] COVID-19-COS: COVID-19 Core Outcomes: An International Initiative to Establish Critically Important Core Outcomes for Trials in COVID-19. 2020 Available at: https://www.covid-19-cos.org. Accessed April 21, 2020

[24] Tong A Craig JC Nagler EV; SONG Executive Committee and the European Renal Best Practice Advisory Board; SONG Executive Committee and the European Renal Best Practice Advisory Board. Composing a new song for trials: The Standardized Outcomes in Nephrology (SONG) initiative. Nephrol Dial Transplant. 2017; 32:1963–1966 29126280 10.1093/ndt/gfx288

[25] Braun V Clarke V Hayfield N Thematic analysis. In: Handbook of Research Methods in Health Social Sciences. 2019 Liamputtong P (Ed). Springer Nature Singapore Pte Ltd. pp 843–960

[26] Parshall MB Schwartzstein RM Adams L; American Thoracic Society Committee on Dyspnea. An official American thoracic society statement: Update on the mechanisms, assessment, and management of dyspnea. Am J Respir Crit Care Med. 2012; 185:435–452 22336677 10.1164/rccm.201111-2042ST PMC5448624

[27] Souto-Miranda S Marques A Triangulated perspectives on outcomes of pulmonary rehabilitation in patients with COPD: A qualitative study to inform a core outcome set. Clin Rehabil. 2019; 33:805–814 30592227 10.1177/0269215518821405

[28] Turnbull AE Sepulveda KA Dinglas VD Core domains for clinical research in acute respiratory failure survivors: An International modified delphi consensus study. Crit Care Med. 2017; 45:1001–1010 28375853 10.1097/CCM.0000000000002435 PMC5433919

